# Alternate-Day Fasting Combined with Exercise: Effect on Sleep in Adults with Obesity and NAFLD

**DOI:** 10.3390/nu15061398

**Published:** 2023-03-14

**Authors:** Mark Ezpeleta, Kelsey Gabel, Sofia Cienfuegos, Faiza Kalam, Shuhao Lin, Vasiliki Pavlou, Krista A. Varady

**Affiliations:** Department of Kinesiology and Nutrition, University of Illinois at Chicago, 1919 West Taylor Street, Room 532, Chicago, IL 60612, USA

**Keywords:** alternate-day fasting, intermittent fasting, non-alcoholic fatty liver disease, hepatic steatosis, sleep quality, sleep duration, insomnia, obstructive sleep apnea

## Abstract

**Objective**: This study investigated how alternate-day fasting (ADF) combined with aerobic exercise impacts body weight and sleep in adults with non-alcoholic fatty liver disease (NAFLD). **Methods**: Adults with obesity and NAFLD (n = 80) were randomized into one of four groups for 3 months: combination of ADF (600 kcal “fast day,” alternated with an ad libitum intake “feast day”) and moderate-intensity aerobic exercise (five sessions per week, 60 min/session); ADF alone; exercise alone; or a no-intervention control group. **Results**: By month 3, body weight and intrahepatic triglyceride content decreased (*p* < 0.001, group × time interaction) in the combination group versus the exercise group and control group, but not versus the ADF group. Sleep quality, measured by the Pittsburgh Sleep Quality Inventory (PSQI), did not change in the combination group (baseline: 6.0 ± 0.7; month 3: 5.6 ± 0.7), ADF group (baseline: 8.9 ± 1.0; month 3: 7.5 ± 0.8), or exercise group (baseline: 6.4 ± 0.6; month 3: 6.7 ± 0.6), versus controls (baseline: 5.5 ± 0.7; month 3: 4.6 ± 0.5). Wake time, bedtime, sleep duration, and insomnia severity did not change (no group x time interaction) over the course of the study in any group. Risk for obstructive sleep apnea was present in 30% of combination subjects, 75% of ADF subjects, 40% of exercise subjects, and 75% of controls, and did not change in the intervention groups, versus controls, by month 3. No associations were observed between changes in body weight, intrahepatic triglyceride content, and any sleep outcome. **Conclusions**: The weight loss induced by ADF combined with exercise does not improve sleep quality, duration, insomnia severity, or risk of obstructive sleep apnea in individuals with NAFLD.

## 1. Introduction

Non-alcoholic fatty liver disease (NAFLD) is defined as the presence of 5% or more fat in the liver, confirmed by hepatic imaging or biopsy [1]. Approximately 25% of adults in the United States are afflicted by NAFLD [2]. Poor sleep may adversely affect insulin sensitivity and inflammatory status [3], thereby contributing to the development and progression of NAFLD. A recent cohort study of nearly 150,000 adults followed over 4 years showed that short sleep duration (≤5 h) was independently associated with an increased risk of incident of NAFLD [4]. In addition, low sleep quality and the presence of obstructive sleep apnea have been shown to exacerbate the severity of NAFLD [5,6]. These findings underscore the importance of healthy sleep in preventing the progression of NAFLD.

Lifestyle interventions that produce 5–10% weight loss have been shown to improve various sleep measures and resolve steatohepatitis [7]. The most commonly implemented lifestyle therapy in patients with NAFLD is daily calorie restriction combined with aerobic exercise. However, more recently, there has been mounting interest in exploring how *intermittent fasting* may benefit people with fatty liver disease. Evidence from two studies show that alternate-day fasting (ADF; 600 kcal “fast day” alternated with an ad libitum intake “feast day”) is effective for reducing liver steatosis score, circulating levels of alanine transaminase (ALT), and body weight in patients with NAFLD [8,9]. In addition, we recently performed a randomized controlled trial showing that ADF combined with aerobic exercise decreased body weight by 5%, intrahepatic triglyceride content by 5%, and ALT concentrations in adults with NAFLD [10]. While these studies are valuable to the field, they are limited in that none of them examined the underlying role of sleep in mediating these effects.

Accordingly, the goal of this study was to investigate how intermittent fasting combined with exercise impacts body weight and sleep measures in adults with NAFLD. We hypothesized that ADF combined with aerobic exercise would produce the greatest weight loss, and in turn, the most pronounced improvements in sleep quality, duration, and insomnia severity, when compared to ADF alone, exercise alone, or controls. 

## 2. Methods

This is a secondary analysis of a 3-month randomized, controlled, parallel-arm study. The trial examined the effects of ADF combined with exercise, to each intervention alone, on intrahepatic triglyceride content and metabolic disease risk factors in patients with NAFLD [10]. Participants were randomized to 1 of 4 intervention groups: ADF plus exercise, ADF alone, exercise alone, or a no-intervention control group. Randomization was performed by a stratified random sampling procedure based on sex, age, BMI, and intrahepatic triglyceride content. The trial was not blinded, but study staff who analyzed the outcome variables were unaware of the participant’s group assignment. 

### 2.1. Subject Selection

Participants were recruited from the University of Illinois Chicago Medical Center. The trial was conducted between January 2020 and March 2022. Subjects were enrolled in four separate rounds (~20 subjects per round). Adults between the ages of 18 and 65 years with a BMI between 30 and 60 kg/m^2^ were screened by survey and ALT blood test. Women with ALT levels greater than 17 U/L and men with ALT levels greater than 25 U/L were admitted for further NAFLD screening by magnetic resonance imaging (MRI). Specifically, their intrahepatic triglyceride (IHTG) content was quantified by magnetic resonance imaging proton density fat fraction (MRI-PDFF). Patients who previously had ultrasound- or biopsy-diagnosed NAFLD also had their diagnosis confirmed by MRI-PDFF. In order to be included in the study, the intrahepatic triglyceride content needed to exceed 5% of liver weight. 

Exclusion criteria were as follows: history of acute or chronic viral hepatitis, autoimmune hepatitis, or drug-induced liver diseases; alcohol consumption greater than 5 alcoholic drinks per week for women and greater than 10 drinks per week for men in the past 6 months; history of diabetes, cardiovascular disease, or chronic kidney disease; weight instability, i.e., more than 4% weight loss/gain in the past 3 months; or a medical condition or injury that would prevent participation in the aerobic training. The protocol was approved by the Office for the Protection of Research Subjects at the University of Illinois at Chicago, and informed consent was obtained from all participants (IRB #2019-0300). This trial was registered at ClinicalTrials.gov (NCT04004403).

### 2.2. Alternate Day Fasting Protocol

As described previously [10], subjects in the combination group and ADF group were asked to eat 600 kcal as a dinner (between 5 and 8 pm) on fast days and eat food ad libitum on alternating feast days. The feast and fast days began at 12 am each day. Therefore, subjects fasted for approximately 17–20 h on the fast day (i.e., from 12 am to 5 pm or 12 am to 8 pm). On each fast day, subjects were asked to consume lots of water and were allowed to drink energy-free beverages such as black coffee, herbal tea, black tea, and sugar-free sodas (max 2 sugar-free sodas per day). Combination group and ADF group subjects were given pre-packaged fast day meals during the first 4 weeks of the study. After this, these subjects received diet counseling to learn how to meet calorie goals on fast days. The pre-packaged fast day meals complied with the American Diabetes Association nutrition guidelines for macronutrient composition (i.e., 30% fat, 55% carbohydrates, and 15% protein). The exercise group and control group were asked to not change their eating habits and did not receive pre-packed foods or any dietary advice during the trial. 

### 2.3. Exercise Protocol

Subjects in the combination group and exercise group performed moderate-intensity aerobic training 5× per week for 3 months. Every exercise session was supervised by the study coordinator. Treadmills, stationary bikes, or elliptical machines were used for the aerobic training. Each training session was performed at the research center. The maximum predicted heart rate (HRmax) was calculated as [(210/min-age)] for women and [(220/min-age)] for men. An activity monitor was used to evaluate Hrmax. Exercise intensity increased over the first 4 weeks of the trial (i.e., from 65 to 80% of Hrmax). The exercise lasted for 60 min per session. Approximately 3 months into the study, the COVID pandemic hit, and subjects transitioned to at-home training using their own exercise equipment. If they did not have any equipment at home, they were instructed to watch aerobic exercise videos on the internet. The training sessions at home were supervised by the study coordinator using video conference platforms. Subjects in the ADF group and control group did not perform the exercise intervention. These subjects were asked not to change their daily activity routines, so as to not confound the study findings. 

### 2.4. Control Group Protocol

Control participants were instructed to maintain their body weight during the 3-month trial by not changing their eating habits or activity routines. The controls received no pre-packaged foods or dietary advice but visited the research center at the same frequency as the other study groups to provide clinical assessments. 

### 2.5. Body Weight, Body Composition, Intrahepatic Triglyceride Content, and Liver Fibrosis

All variables were assessed at baseline and month 3. Body weight was assessed without shoes, in light clothing, using a digital scale (HealthOMeter) at the research center. Height was assessed using a wall-mounted stadiometer. BMI was calculated as kg/m^2^. Fat mass, lean mass, and visceral fat mass were measured after an 8-hour fast by dual X-ray absorptiometry (iDXA, GE). 

Intrahepatic triglyceride content was measured by MRI-PDFF [10]. These scans were carried out at the UIC Center for Magnetic Resonance Research. A SIEMENS 3.0-Tesla MRI scanner was used for the baseline and month 3 liver fat estimations. T1 volumetric interpolated breath-hold examination (VIBE) Dixon sequence was used to obtain fat–water separation images. The following parameter settings were employed: TE1 = 2.5 ms; TE2 = 3.7 ms; repetition time = 5.47 ms; 5° flip angle; ± 504.0 kHz per pixel receiver bandwidth; slice thickness = 3.0 mm. Irregular-shaped regions of interest covering the entire liver were used to quantify liver fat content. MRIs were performed by a trained radiologist for each subject. MRI-PDFF maps were generated by placing circular ROIs with diameters of 20 mm centrally in each of the liver segments. The average fat content values were calculated for the entire liver. 

The degree of liver fibrosis was estimated using the Fibrosis-4 (FIB-4) index, as follows: Age (years) × AST (IU/L)/(√ALT (IU/L) × Platelet count (10^9^/L)) [11]. A FIB-4 score below 1.30 is an indicator for low risk for advanced fibrosis, while a score above 2.67 is an indicator for high risk for advanced fibrosis.

### 2.6. Energy Intake and Physical Activity

Energy intake was assessed by the National Cancer Institute (NCI) web-based system, Automated Self-administered 24-hour Dietary Assessment Tool (ASA24), over 7 days at baseline and month 3. Habitual physical activity (not including the aerobic exercise program) was measured by a pedometer (Fitbit Alta) worn for 7 days at baseline and at month 3. 

### 2.7. Sleep Measures

Sleep quality, duration, and timing were measured using the Pittsburgh Sleep Quality Index (PSQI) [12]. The PSQI is a self-report survey with 19-items that measures sleep quality in the past month, resulting in a total score of 0–21. Scores above 5 can be used as an indicator of poor sleep quality. Insomnia severity was measured by the Insomnia Severity Index (ISI) [13]. The ISI is a 7-item self-report questionnaire that rates each item by a 5-point Likert scale. The ISI produces a total score of 0–28 points. Scores fall into the following categories: no clinically significant insomnia (score of 0–7); subthreshold insomnia (score of 8–14); moderate-severity insomnia (score of 15–21); and severe insomnia (score of 22–28). The risk of obstructive sleep apnea (% occurrences) was estimated in all subjects by the 10-item self-report Berlin Questionnaire [14].

### 2.8. Statistical Analysis

All data are presented as means ± SEM. At baseline, differences between groups were tested by one-way ANOVA (continuous variables) or the McNemar test (categorical variables). Repeated-measures ANOVA with groups (combination, ADF, exercise, and control) as the between-subject factor and time (baseline and month 3) as the within-subject factor was used to compare changes in dependent variables between the groups over time. Pearson correlations were performed to assess the relationships between changes in body weight, intrahepatic triglyceride content, and sleep measures. Differences were considered significant at *p* < 0.05. All data were analyzed using SPSS software (version 27, SPSS Inc., Chicago, IL, USA).

## 3. Results

### 3.1. Subject Baseline Characteristics and Dropouts

As previously reported [10], 132 individuals were assessed for eligibility, and 52 of these individuals did not meet one or more of the inclusion criteria. A total of 80 participants were randomized into the combination group (n = 20), ADF group (n = 20), exercise group (n = 20), or the control group (n = 20). The number of completers were as follows: combination group, n = 20; ADF group, n = 19; exercise group, n = 15; and control group, n = 20. 

Table 1 displays the baseline characteristics of the participants. At baseline, there were no significant differences between groups for body weight, body composition, intrahepatic triglyceride content, liver fibrosis score, energy intake, physical activity, or any sleep variable. 

### 3.2. Body Weight, Body Composition, Intrahepatic Triglyceride Content, and Liver Fibrosis

**Figure 1:**
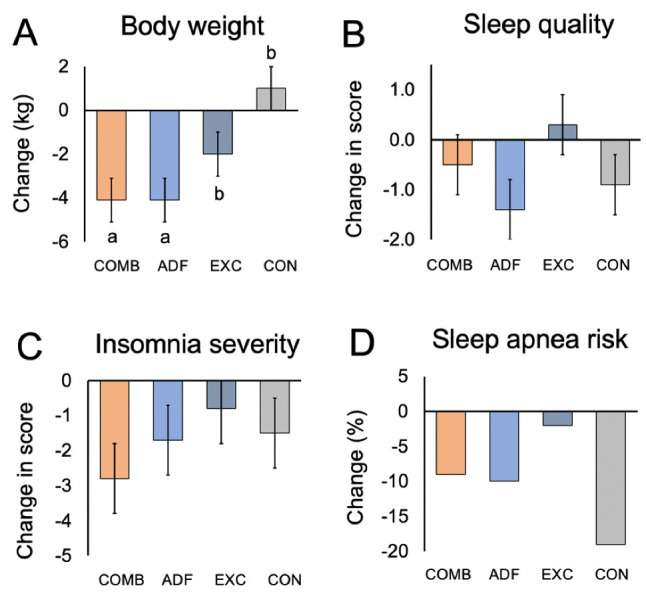
**Change in body weight, sleep quality, insomnia severity, and risk of obstructive sleep apnea after 3 months of intervention.** Continuous variables reported as mean ± SEM. Risk of obstructive sleep apnea reported as % occurrences. Comb: combination group; ADF: alternate-day fasting group; Exc: exercise group; Con: control group. Means with different superscript letters are significantly different using repeated measures ANOVA (*p* < 0.05, group × time interaction). (**A**) By month 3, body weight decreased (*p* < 0.01, group × time interaction) in the combination group versus the exercise group and controls, but not versus the ADF group. (**B**) Sleep quality (PSQI) score did not change in the intervention groups versus controls (no group × time interaction). (**C**) Insomnia severity did not change in the intervention groups versus controls (no group × time interaction). (**D**) Risk of obstructive sleep apnea did not change in the intervention groups versus controls (no group × time interaction).

By month 3, body weight decreased (*p* < 0.01, group x time interaction) in the combination group versus the exercise group and controls, but not versus the ADF group (Table 1, Figure 1A). Likewise, fat mass was reduced (*p* = 0.02, group × time interaction) in the combination group versus the exercise group and controls, but not versus the ADF group (Table 1). Lean mass, visceral fat mass, and BMI did not change significantly between groups. By month 3, intrahepatic triglyceride content decreased (*p* = 0.02, group × time interaction) in the combination group versus the exercise group and controls, but not versus the ADF group (Table 1). The liver fibrosis score did not change significantly between groups by month 3.

### 3.3. Energy Intake and Physical Activity

By month 3, energy intake decreased (*p* < 0.05, group x time interaction) in the combination group versus the exercise group and controls, but not versus the ADF group (Table 1). Regular physical activity (excluding the exercise intervention program) did not change in any of the groups over time (Table 1).

### 3.4. Sleep Measures

Sleep quality, timing, and duration were measured by the PSQI survey. A PSQI total score greater than 5 indicates poor sleep quality. At the beginning of the study, the average scores for PSQI were 6.0 ± 0.7 for the combination group, 8.9 ± 1.0 for the ADF group, 6.4 ± 0.6 for the exercise group, and 5.5 ± 0.7 for controls, indicating poor sleep quality in all groups at baseline (Table 1). After 3 months, the sleep quality scores did not change significantly (no group × time interaction) in any intervention group, relative to controls (Figure 1B). Wake time, bedtime, and sleep duration did not change (no group × time interaction) over the course of the study in any group (Table 1). By month 3, the insomnia severity scores did not change significantly (no group x time interaction) in the intervention groups, relative to controls (Table 1, Figure 1C). The risk for obstructive sleep apnea was present in 30% of combination subjects, 75% of ADF subjects, 40% of exercise subjects, and 75% of controls, and at baseline (Table 1). By the end of the trial, the risk of obstructive sleep apnea did not change in the intervention groups versus controls (Figure 1D). There were no associations between changes in sleep quality, duration, or insomnia severity and changes in body weight, intrahepatic triglyceride content, or liver fibrosis.

## 4. Discussion

This is the first study to examine the effects of intermittent fasting combined with aerobic exercise on sleep in adults with NAFLD. Our results show that this combination intervention produced significant reductions in body weight and intrahepatic triglyceride content but no changes in sleep quality, duration, insomnia severity, or risk of obstructive sleep apnea.

Weight loss by dietary restriction may improve sleep quality and duration by reducing sleep fragmentation and alleviating sleep-disordered breathing [15,16]. In the present study, the combination of ADF and aerobic exercise produced significant reductions in body weight (~5%) and liver fat (~5%), but no change in sleep quality or duration in adults with NAFLD. 

Our findings are complementary to those of other fasting trials showing no impact on these sleep measures. For instance, in the trial by Kalam et al. [17], 6 months of ADF combined with a high-protein/low-carbohydrate diet produced 6% weight loss but no change in sleep quality or duration in participants with obesity. Likewise, Gabel et al. [18] and Cienfuegos et al. [19] reported no change in sleep quality or duration after 2–3 months of time-restricted eating, despite 3% weight loss. Moreover, Wilkinson et al. [20] demonstrated no change in sleep quality after 2 months of time-restricted eating, even though their participants reduced body weight by 4%. In contrast, studies examining the impact of aerobic exercise on sleep quality and duration in patients with obesity generally report improvements, even in the absence of significant weight loss [21,22]. 

There are several reasons why sleep quality and duration may not have improved in our trial. First, our participants were on the cusp of being “good sleepers” at baseline (based on PSQI scores [12]). If their sleep quality was worse at the beginning of the study, we may have been more likely to observe improvements. Second, the study was conducted during the COVID-19 pandemic, and the stress induced by lockdown conditions may have had detrimental effects on sleep quality and duration [23]. Lastly, our participants were averaging 7.5 h of sleep per night, which is in line with what is considered healthy by the National Sleep Foundation [24]. 

Changes in insomnia severity were also assessed. At baseline, participants in the combination and ADF groups portrayed sub-clinical insomnia (ISI score 8–14), while subjects in the exercise and control groups displayed no clinically significant insomnia (ISI score 0–7). By the end of the trial, no significant changes in insomnia scores were noted in the intervention groups versus controls. This finding is not surprising as our subjects did not portray clinically significant insomnia at baseline; thus, it would be unlikely for this sleep measure to improve. These findings are in accordance with other trials of ADF [17], time-restricted eating [18,19], and aerobic exercise [25], which show no change in insomnia severity in those who are not afflicted by this condition.

The risk of obstructive sleep apnea did not change during the trial. At the beginning of the study, close to half of our cohort (~50%) was at high risk of obstructive sleep apnea. While we observed that the risk for sleep apnea decreased *numerically* in all the intervention groups, these changes were not significant relative to controls. However, it is possible that our interventions did not achieve enough weight reduction to improve this sleep metric. Accumulating evidence suggests that at least 10% weight loss may be necessary to decrease the risk of obstructive sleep apnea in people with obesity [26]. 

This study has several limitations. First, our sample size was small (i.e., n = 80 in total, n = 20 per group). Moreover, our power calculation was based solely on intrahepatic triglyceride content, so it is likely that this study was not powered adequately to identify significant changes in sleep parameters, such as the insomnia and PSQI score. Second, all sleep outcomes were quantified via self-report. This study would have benefitted from the use of wrist actigraphy to provide more objective assessments of rest and activity patterns. Third, this study was conducted during the coronavirus pandemic, which most likely impacted our participants’ daily routines and regular sleep habits [23]. Fourth, the trial duration was short (3 months). Thus, the longer-term effects of intermittent fasting, alone or combined with exercise on sleep parameters, remain unknown. Fifth, the degree of weight loss produced by the combination and ADF interventions was moderate and fell short of being clinically significant (i.e., >5% weight loss from baseline). Lastly, subjects were permitted to drink caffeinated beverages during their fasting window. As such, some participants may have consumed caffeine late into the evening, which may have impacted their sleep.

In summary, these findings suggest that the weight loss induced by ADF combined with exercise does not improve sleep quality, duration, insomnia severity or risk of obstructive sleep apnea in individuals with obesity and NAFLD. However, these findings will need to be confirmed by a well-powered randomized controlled trial specifically designed to assess the impact of these lifestyle interventions on sleep in this population group.

## Figures and Tables

**Table 1:** Body weight, body composition, and sleep variables after 3 months of intervention.

	Combination		ADF		Exercise		Control	
	Baseline	Month 3	Baseline	Month 3	Baseline	Month 3	Baseline	Month 3
**Demographics**								
n	20	20	20	19	20	15	20	20
Age	44 ± 3	--	44 ± 3	--	44 ± 3	--	44 ± 3	--
Sex (Female/Male)	17/3	--	16/4	--	16/4	--	16/4	--
**Body weight**								
Body weight (kg)	101 ± 4	97 ± 4 ^a^	96 ± 5	92 ± 4 ^a^	100 ± 5	99 ± 5 **^b^**	100 ± 5	99 ± 5 **^b^**
Fat mass (kg)	46 ± 3	43 ± 3 ^a^	40 ± 2	37 ± 3 ^a^	45 ± 3	43 ± 11 **^b^**	45 ± 3	44 ± 11 **^b^**
Lean mass (kg)	51 ± 3	51 ± 3	51 ± 2	49 ± 2	52 ± 3	51 ± 3	51 ± 2	50 ± 3
Visceral fat (kg)	1.6 ± 0.2	1.4 ± 0.2	1.6 ± 0.2	1.4 ± 0.1	1.7 ± 0.2	1.7 ± 0.2	1.7 ± 0.2	1.7 ± 0.2
BMI (kg/m^2^)	37 ± 5	35 ± 5	36 ± 8	34 ± 8	37 ± 6	36 ± 5	37 ± 5	37 ± 6
**Liver parameters**								
IHTG (%)	18 ± 2	13 ± 1 ^a^	16 ± 2	14 ± 1 ^a^	17 ± 2	16 ± 2 **^b^**	17 ± 3	17 ± 3 **^b^**
Fibrosis score	0.91 ± 0.08	0.75 ± 0.06	0.93 ± 0.13	0.85 ± 0.10	0.86 ± 0.08	0.80 ± 0.07	0.76 ± 0.05	0.75 ± 0.05
**Energy intake (kcal/d)**	2062 ± 287	1356 ± 162 ^a^	1940 ± 132	1285 ± 130 ^a^	1808 ± 170	1779 ± 175 **^b^**	1810 ± 132	1833 ± 150 **^b^**
**Steps/d**	7434 ± 943	7455 ± 942	7528 ± 1023	7041 ± 882	6754 ± 611	6936 ± 619	6497 ± 652	6004 ± 577
**PSQI**								
Total score	6.0 ± 0.7	5.6 ± 0.7	8.9 ± 1.0	7.5 ± 0.8	6.4 ± 0.6	6.7 ± 0.6	5.5 ± 0.7	4.6 ± 0.5
Wake time (h:min)	6:30 ± 0:15	7:15 ± 0:30	6:20 ± 0:25	6:00 ± 0:20	6:15 ± 0:15	6:30 ± 0:20	6:15 ± 0:15	6:10 ± 0:15
Bedtime (h:min)	22:50 ± 0:20	23:15 ± 0:20	22:40 + 0:20	22:40 ± 0:20	23:00 ± 0:15	22:45 ± 0:10	22:00 ± 0:50	22:40 ± 0:15
Sleep duration (h)	7.6 ± 1.2	8.0 ± 1.2	7.8 ± 1.7	7.3 ± 1.7	7.3 ± 1.0	7.7 ± 1.1	8.1 ± 1.4	7.5 ± 1.2
**Insomnia severity (ISI)**								
Total score	9.2 ± 1.5	6.4 ± 1.3	8.1 ± 1.2	6.4 ± 0.9	7.4 ± 0.9	6.7 ± 0.8	6.4 ± 1.1	4.9 ± 0.9
**Berlin questionnaire**								
Risk obstructive sleep apnea	30%	21%	75%	65%	40%	38%	75%	56%

Continuous variables reported as mean ± SEM. Risk of obstructive sleep apnea reported as % occurrences. ADF: Alternate day fasting, BMI: Body mass index, IHTG: Intrahepatic triglyceride content, PSQI: Pittsburg Sleep Quality Index, ISI: Insomnia severity index. At baseline, no significant differences between groups for any parameter (ANOVA for continuous variables; McNemar test for categorical variables). Means with different superscript letters are significantly different using repeated measures ANOVA (*p* < 0.05, group × time interaction).

## Data Availability

Data is unavailable due to privacy or ethical restrictions.
